# Discrimination and Depressive Symptoms: The Role of Psychological Resilience across Racial and Ethnic Groups

**DOI:** 10.1093/geroni/igaf122.3048

**Published:** 2025-12-31

**Authors:** Yi Wang, Yifan Lou, Huei-wern Shen, Man Guo

**Affiliations:** University of Iowa School of Social Work, Iowa City, Iowa, United States; Virginia Commonwealth University, Richmond, Virginia, United States; University of North Texas, Denton, Texas, United States; University of Iowa, Iowa City, Iowa, United States

## Abstract

Despite extensive research on the detrimental effects of discrimination on health, less is known about how multi-dimensional discrimination experiences affect mental health and the potential role of psychological resilience across racial/ethnic groups. To address these gaps, this study analyzed data from 11,833 respondents in the 2016/2018 Health and Retirement Study Psychosocial Leave-Behind Questionnaire (65% non-Hispanic White, 18% non-Hispanic Black, 13% Hispanic, and 4% Other). Respondents reported on six forms of discrimination: disrespect, poor service in restaurants, poor treatment from doctors, perceived as unintelligent, fear from others, and threats. Psychological resilience, defined as the ability to navigate adversity through positive adaption, was assessed using a 12-item index covering domains such as perseverance despite major setbacks, adaptability to change, and recognition of inner strengths. Latent class analysis identified three distinct typologies: (C1) few experiences (70%)—low across all experiences, (C2) microaggressions (25%)—high on disrespect, poor service at restaurant, and perceived as unintelligent, and (C3) intensive discrimination (5%)—high across all experiences. Structural equation modeling revealed that for non-Hispanic Whites and Blacks, C2 and C3 were associated with higher depressive symptoms, both directly and indirectly via lower psychological resilience. Among Hispanics, resilience mediated the effects of microaggressions (C2) on depressive symptoms, while for Other, this mediation effect was observed for intensive discrimination (C3). The findings highlight discrimination as a mental health risk factor for older Americans across racial/ethnic groups and suggest that psychological resilience may mitigate its impact, contingent on experience type and racial/ethnic background. Implications for future research and practice are discussed.

